# Regional inequities in acute stroke care in Norway: a national benchmark for the “stroke action plan for Europe” implementation

**DOI:** 10.1093/esj/aakag072

**Published:** 2026-06-29

**Authors:** Martin W Kurz, Elisa de la Fuente Sanz, Ivy Alo, Isaac Alo, Mehdi Rezai, Kathinka D Kurz, Melinda B Roaldsen, Agnethe Eltoft, Hilde Karen Ofte, Fredrik Ildstad, Annette Fromm, Arnstein Tveiten, Guri Hagberg, Marianne Altmann, Soffien Ajmi

**Affiliations:** Department of Neurology and Neuroscience Research Group, Stavanger University Hospital, Stavanger, Norway; Department of Clinical Science, University of Bergen, Bergen, Norway; Department of Neurology and Neuroscience Research Group, Stavanger University Hospital, Stavanger, Norway; Department of Neurology and Neuroscience Research Group, Stavanger University Hospital, Stavanger, Norway; Department of Clinical Science, University of Bergen, Bergen, Norway; Department of Neurology and Neuroscience Research Group, Stavanger University Hospital, Stavanger, Norway; Department of Clinical Science, University of Bergen, Bergen, Norway; Department of Neurology and Neuroscience Research Group, Stavanger University Hospital, Stavanger, Norway; Department of Radiology and Stavanger Medical Imaging Laboratory, Stavanger University Hospital, Stavanger, Norway; Department of Electrical Engineering and Computer Science, University of Stavanger, Stavanger, Norway; Centre of Digitalisation, Development and Integrative Care, University Hospital of North Norway, Tromsø, Norway; Department of Clinical Medicine, UiT The Arctic University of Norway, Tromsø, Norway; Department of Clinical Medicine, UiT The Arctic University of Norway, Tromsø, Norway; Department of Neurology, University Hospital of North Norway, Tromsø, Norway; Department of Neurology, Nordland Hospital Trust, Bodø, Norway; Department of Neuromedicine and Movement Science, Faculty of Medicine and Health Sciences, Norwegian University of Science and Technology (NTNU), Trondheim, Norway; Stroke Unit, Department of Medicine, Trondheim University Hospital, Trondheim, Norway; Department of Neurology, Haukeland University Hospital, Bergen, Norway; Department of Neurology, Hospital of Southern Norway, Kristiansand, Norway; Stroke Unit, Department of Neurology, Oslo University Hospital, Ullevål, Norway; Department of Medical Research, Bærum Hospital Vestre Viken Hospital Trust, Drammen, Norway; Department of Neurology, Akershus University Hospital, Lørenskog, Norway; Department of Neurology and Neuroscience Research Group, Stavanger University Hospital, Stavanger, Norway

**Keywords:** acute ischaemic stroke, CT perfusion, EVT, health services accessibility, intravenous thrombolysis, magnetic resonance imaging, reperfusion therapy, regional variation, stroke care organisation, stroke networks

## Abstract

**Introduction:**

The Stroke Action Plan for Europe (SAP-E) calls for equitable access to evidence-based acute stroke care, yet within-country variation remains underexplored. We mapped reperfusion capabilities, imaging resources and organisational structures across all Norwegian hospitals providing acute stroke care.

**Patients and methods:**

In 2024, we conducted a cross-sectional survey of all 50 acute stroke hospitals. Data covered reperfusion protocols, advanced imaging availability and the organisational maturity of regional stroke networks. Hospitals were categorised by their most advanced routinely available strategy: intravenous thrombolysis (IVT) within 0–4.5 h (IVT ≤ 4.5 h); IVT in wake-up stroke; IVT in both wake-up stroke and the extended window or endovascular thrombectomy (EVT) capability.

**Results:**

All hospitals providing local acute stroke treatment offered IVT ≤ 4.5 h, while 3 hospitals in South-Eastern Norway had a stroke unit but transferred all acute patients directly to a comprehensive stroke centre. Eleven hospitals (22%) provided IVT ≤ 4.5 h only, 8 (16%) implemented IVT for wake-up stroke and 28 (56%) offered IVT for both wake-up and extended-window thrombolysis. Eight hospitals (16%) were EVT-capable, unevenly distributed. CT perfusion (CTP) was available in 28 hospitals (56%), and acute MRI in 17 (34%). Regional stroke networks varied widely in structure and activity, from well-organised, funded systems to inactive or absent networks.

**Conclusion:**

Norway shows universal IVT access within 0–4.5 h but marked regional variation in advanced treatment, imaging and coordination. Strengthening and standardising regional stroke networks may be key to achieving SAP-E’s vision of equitable, high-quality care.

## Graphical Abstract

**Graphical Abstract:**
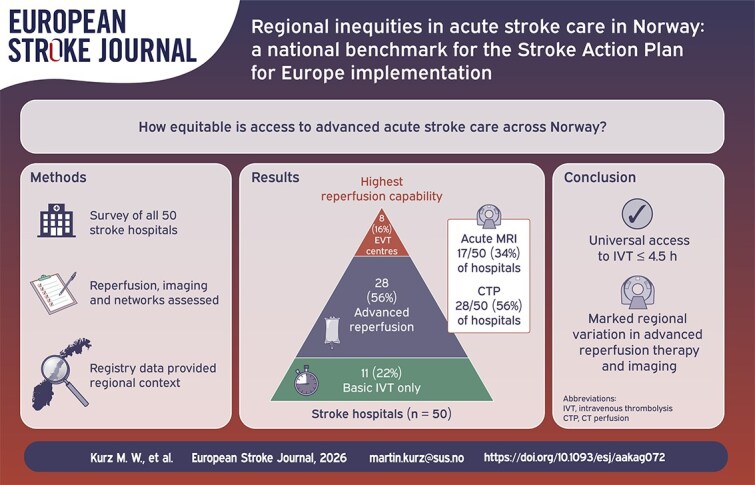

## Introduction

Stroke remains a leading global cause of mortality and long-term disability, contributing to over 7 million deaths annually and placing a heavy burden on healthcare systems and societies worldwide.^1^ In Norway, around 10,000–11,000 individuals are hospitalised annually with suspected acute stroke, a number expected to rise as the population ages and more patients survive their initial stroke, increasing the pool at risk for recurrent events.^2^^,^^3^

Over the past decade, major advances in acute stroke treatment—including endovascular thrombectomy (EVT), wake-up stroke treatment and extended time window thrombolysis—have expanded treatment opportunities beyond traditional therapeutic windows.^4–7^ Successful implementation of these interventions requires access to advanced neuroimaging, specialised expertise and streamlined workflows.^8^^,^^9^ Consequently, access to and adoption of these treatments can vary between hospitals, creating the potential for regional disparities in health care.

Norway has a population of about 5.5 million, served by 50 hospitals providing acute stroke treatment, distributed across large geographic distances and 4 regional health trusts.^2^ National clinical guidelines aim to ensure high-quality and equitable care,^10^ yet practical implementation in Norway faces barriers such as uneven distribution of EVT services, limited availability of magnetic resonance imaging (MRI) and computed tomography perfusion (CTP) imaging, shortages of stroke specialists and inconsistent application of advanced protocols. Similar organisational and geographic challenges have also been described internationally, particularly in rural or resource-constrained settings.^11^

The *Stroke Action Plan for Europe* (*SAP-E*) highlights the need to identify and address within-country variations in stroke care, including differences by geographical region, as a foundation for equitable service provision.^12^ It also calls for strengthening regional stroke networks and developing comparable data systems to monitor quality and outcomes.

The objective of this study was therefore to map hospital-level disparities in diagnostic and therapeutic stroke capabilities for acute ischaemic stroke across Norway, classifying hospitals according to treatment availability (intravenous thrombolysis [IVT] within the 0–4.5 h window [IVT ≤ 4.5 h], IVT in wake-up stroke, IVT in the extended-window and EVT), neuroimaging resources and organisational structures. This study focused on hospitals providing acute treatment for ischaemic stroke. By linking these findings to the SAP-E priorities on regional variation, network development and benchmarking, this work aims to provide both a national baseline and a methodological model that could be applied in other countries to support the SAP-E implementation agenda.

## Materials and methods

### Study design and setting

This national, cross-sectional study was conducted between September and October 2024 to assess the diagnostic and therapeutic capabilities of Norwegian hospitals providing acute stroke treatment. The analysis reflects the status of stroke care infrastructure at the end of 2024, acknowledging that hospital capabilities may evolve over time. The aim was to provide a comprehensive, point-in-time overview across all health regions in Norway, using a structured approach that could be replicated in other countries to support international comparisons. The study focused on hospital-level infrastructure and organisation; population-level stroke incidence and admission volumes from national registry data were used to provide regional context.

### Participants

All 50 hospitals in Norway with acute stroke care services were invited to participate. These hospitals were identified through the Norwegian Stroke Registry, a mandatory national quality registry, and confirmed through regional network affiliations and the study team’s familiarity with the national stroke service structure. The study was designed to capture hospital-level stroke care capacity in the context of the populations served by each health region, complementing patient-level outcome data reported in the Norwegian Stroke Registry.

### Data collection

Structured telephone interviews were conducted with key informants at each hospital, including stroke coordinators, stroke physicians and department heads involved in acute stroke management. A standardised registration form, specifically developed for this study, guided all interviews.

The form collected information on:

- Treatment availability: IVT ≤ 4.5 h, IVT in wake-up stroke, IVT in the extended window and EVT.
- Imaging infrastructure: access to computed tomography (CT), CT angiography (CTA), and CTP, MRI and acute MRI availability.
- Organisational resources: 24/7 stroke team availability (defined as a structured stroke-specific call service staffed by stroke specialists, separate from general consultant on-call systems).
- Stroke network organisation: structure and collaboration within regional stroke networks, including referral pathways, inter-hospital coordination and use of teleradiology or teleconsultation systems.

Hospital-level survey data were complemented with publicly available aggregated data from the Norwegian Stroke Registry to provide contextual information on regional stroke volumes and population-level activity. Registry data were not used to evaluate individual hospital performance but to support regional comparisons and interpretation of service availability.

### Validation and quality assurance

Following the interviews, collected data were reviewed and validated by board members of the Norwegian Stroke Organisation to ensure accuracy and consistency across regional responses. In cases where information was incomplete or unclear, hospitals were re-contacted via telephone or email for clarification.

### Anonymisation

To protect institutional confidentiality, data are reported in aggregate at the regional level. No individual hospitals are named or otherwise identifiable, in accordance with ethical standards for publication and data protection.

### Definitions

For the purposes of this study, the following terms were applied consistently:

- IVT ≤ 4.5 h: standard alteplase or tenecteplase treatment within the conventional 4.5-h window.
- IVT in wake-up stroke: IVT based on imaging selection (MRI DWI/FLAIR mismatch or CTP mismatch) in patients with unknown time of onset.
- IVT in the extended window: IVT guided by advanced imaging between 4.5 and 9 h after last known well.
- EVT: catheter-based removal of large vessel occlusion within guideline-recommended time windows.
- Comprehensive stroke centre (CSC): hospital with EVT capability, advanced imaging (CTP and/or MRI) and specialised stroke expertise.
- Stroke unit: dedicated ward for acute stroke care providing organised multidisciplinary treatment, with or without advanced reperfusion services.
- Dedicated 24/7 stroke team: a structured on-call service specifically for acute stroke, staffed by stroke-trained physicians, distinct from the hospital’s general neurology or internal medicine on-call system.

### Hospital classification criteria

Hospitals were categorised based on:

- Treatment scope: IVT ≤ 4.5 h, IVT in wake-up stroke and/or IVT in the extended window, and/or EVT.
- Imaging capabilities: particularly the availability of CTA, CTP and acute MRI.
- Support structures: availability of a dedicated 24/7 stroke team (distinct from general on-call services), local implementation of stroke treatment protocols and participation in organised stroke networks.
- Hospitals were grouped by their respective health regions:
  - Northern Norway (Helse Nord)
  - Central Norway (Helse Midt)
  - Western Norway (Helse Vest)
  - South-Eastern Norway (Helse Sør-Øst)

### Data analysis

Descriptive statistics were used to summarise stroke care capacity and service characteristics across hospitals. Hospitals were mapped based on their diagnostic and treatment capabilities to visualise geographic disparities in access to advanced stroke care. Key comparisons included the distribution of EVT services, access to advanced imaging and protocol implementation across health regions.

### Ethics

The study was based on a structured telephone survey of hospitals and regional stroke network representatives. Participation was voluntary and informed. No patient or individual-level data were collected, and no interventions were performed. According to Norwegian regulations, such institutional surveys do not require approval from a regional ethics committee.

## Results

### Treatment capabilities

All 50 hospitals providing acute stroke care in Norway participated in the survey. Three hospitals in South-Eastern Norway functioned as “one-door-in” sites, transferring all acute stroke patients directly to CSCs and therefore not providing local IVT. Among the remaining 47 hospitals providing local thrombolysis, 28 hospitals (56%) offered IVT across all recommended time windows, including IVT ≤ 4.5 h, IVT for wake-up stroke and IVT in the extended window. A further 8 hospitals (16%) provided IVT limited to the conventional ≤ 4.5-h window and/or wake-up stroke protocols, while 11 hospitals (22%) offered IVT only within the ≤ 4.5-h window. Eight hospitals (16%) were EVT-capable, with marked geographic clustering.

**Figure 1:**
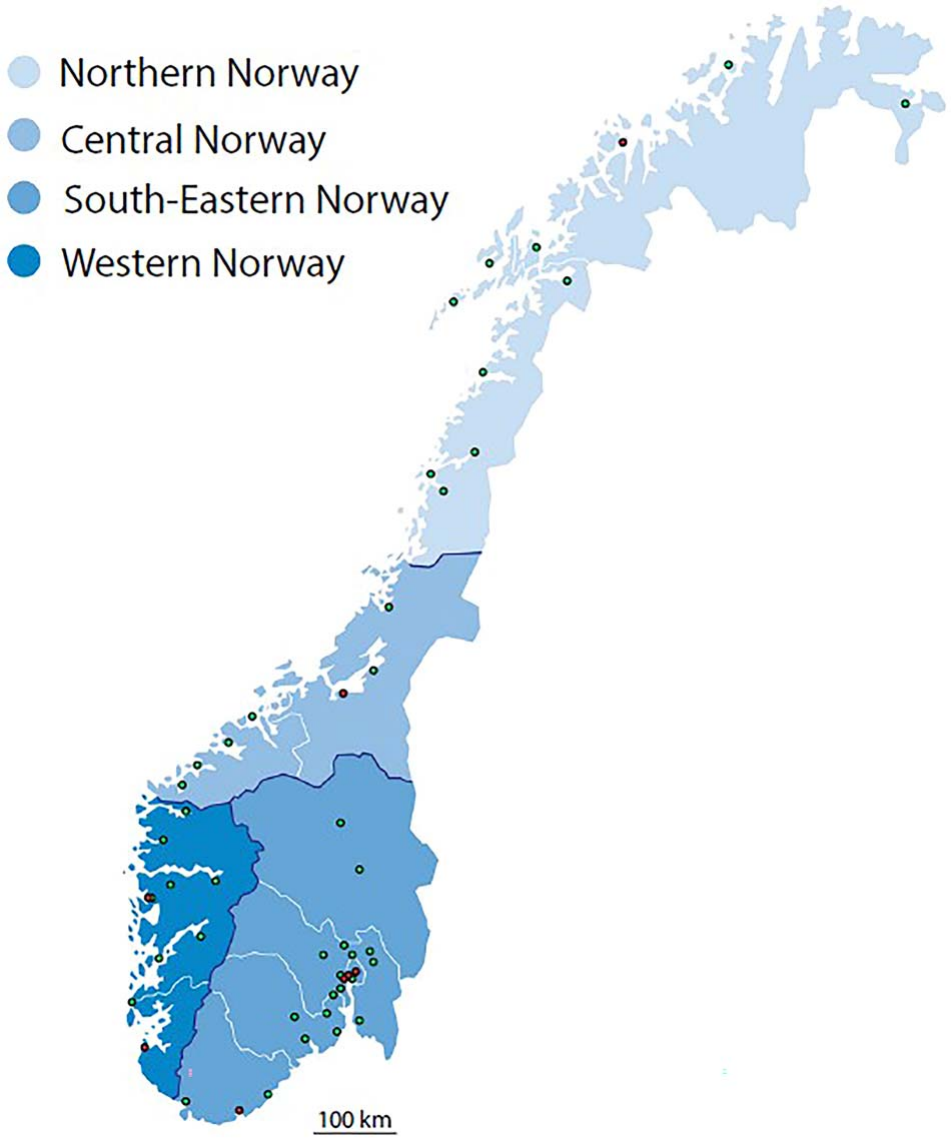
Geographic distribution of hospitals providing acute stroke care in Norway. CSC with EVT capability are shown in red, and stroke units without local EVT coverage are shown in green. Regional health authority boundaries (Northern, Central, Western and South-Eastern Norway) are indicated. Abbreviations: CSC = comprehensive stroke centres, EVT = Endovascular thrombectomy.

In several sites, technical capacity for advanced reperfusion protocols existed but was not routinely applied, mainly due to limited stroke staffing, insufficient 24/7 radiology coverage or absence of formalised procedures (Table 1). The geographic distribution of hospitals and their reperfusion capabilities is shown in Figure 1, highlighting substantial travel distances to CSCs across all health regions.

**Table 1:** Acute stroke treatment and imaging capacity in Norwegian hospitals by health region.

Region	Hospitals	EVT centres	IVT ≤ 4.5 h	IVT ≤ 4.5 h + wake up	IVT ≤ 4.5 h + wake up + extended	CTP available	Acute MRI available	Dedicated 24/7 stroke call team
**Northern Norway**	11	1	5	4	2	4	1	0
**Central Norway**	7	1	0	0	7	6	2	1
**Western Norway**	10	2	5	0	5	5	2	1
**South-Eastern Norway**	22	4	1	4	14	13	12^a^	2

Abbreviations: CTP = CT perfusion; MRI = magnetic resonance imaging; IVT = intravenous thrombolysis; EVT = endovascular thrombectomy.

Data are presented for all hospitals providing acute stroke care (*n* = 50), grouped by health region. Hospitals are categorised according to the most advanced reperfusion treatment provided: IVT within the 0–4.5 h window (IVT ≤ 4.5 h); IVT in wake-up stroke protocols and IVT in wake-up stroke and extended-window protocols. Availability of CTP, acute MRI and dedicated 24/7 stroke call teams is reported. Numbers represent hospitals within each category.

^a^Includes 2 hospitals where acute MRI is available only during weekdays.

### Stroke volume and reperfusion treatment by region

In 2024, a total of 8845 patients with acute stroke were registered in the Norwegian Stroke Registry, corresponding to an estimated national coverage of 86%. Of these, 5022 were treated in South-Eastern Norway, 1443 in Western Norway, 1398 in Central Norway and 982 in Northern Norway. Nationally, 51% of patients with acute cerebral infarction were admitted within 4 h of symptom onset in 2024. At the regional level, differences were modest, with slightly lower proportions in Northern and Western Norway (47%–51%) compared with Central and South-Eastern Norway (52%–55%), consistent with longer prehospital transport distances and more dispersed populations.

### Regional variation

**Figure 2:**
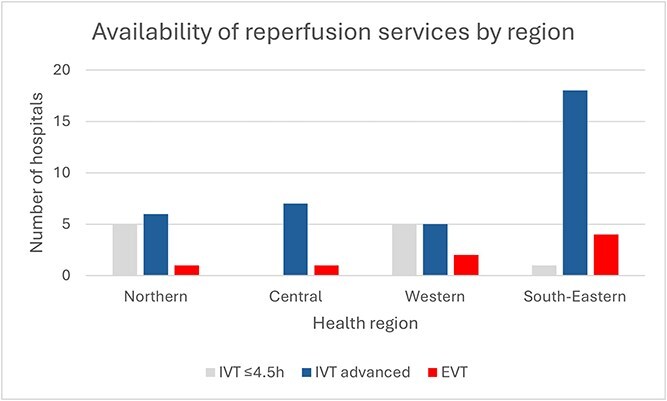
Availability of reperfusion services across Norwegian health regions. Bars show the number of hospitals in each region providing IVT limited to the 0–4.5 h window, IVT including wake-up and/or extended-window protocols (IVT advanced) and EVT. Hospitals are classified according to their highest level of reperfusion capability. Data correspond to those presented in Table 1. Abbreviations: IVT = intravenous thrombolysis, EVT = endovascular thrombectomy.

Availability of reperfusion services varied substantially across health regions, with a higher concentration of hospitals providing advanced IVT protocols and EVT in Central and South-Eastern Norway compared with Northern and Western regions (Figure 2).

**Figure 3:**
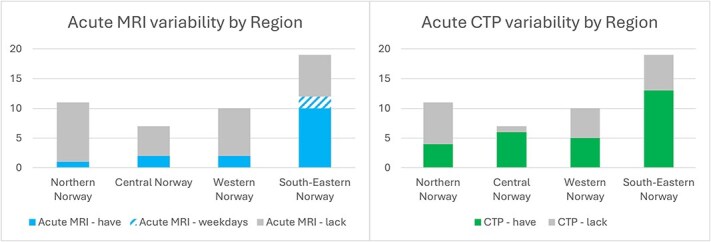
Availability of advanced imaging across Norwegian health regions. Bars show the number of hospitals in each region (Y-axis) with (coloured) and without (grey) access to CT perfusion (CTP, right panel) and acute MRI (left panel). Hospitals with weekday-only MRI access are included as “with access.” Three hospitals in South-Eastern Norway functioned as one-door-in sites, transferring all suspected acute stroke patients to a comprehensive stroke centre for initial imaging and treatment, and were therefore excluded from the denominator. Data correspond to those presented in Table 1. Abbreviation: CTP = CT perfusion.

Northern Norway (11 hospitals), serving approximately 488,000 inhabitants across Nordland, Troms and Finnmark, treated 982 registered acute stroke cases in 2024 according to the Norwegian Stroke Registry. The region had one EVT centre with advanced imaging, yet without 24/7 availability of wake-up stroke and extended-window thrombolysis. Of the 10 remaining stroke units, 6 had wake-up protocols and 2 routinely performed extended-window thrombolysis, mostly during weekday hours. Five provided IVT ≤ 4.5 h. CTP was available in 4 sites, acute MRI only at the EVT centre (outside regular working hours). None of the hospitals had a dedicated 24/7 stroke call team (Figure 3).

Central Norway (7 hospitals), covering approximately 750,000 inhabitants in Trøndelag and parts of Møre and Romsdal, reported 1398 acute stroke admissions in 2024 in the Norwegian Stroke Registry. One EVT centre serves as the regional hub. All 6 stroke units routinely offer IVT ≤ 4.5 h, IVT in wake-up stroke and IVT in extended-window, with CTP lacking at only one site (Figure 3). Acute MRI was available at 1 stroke unit in addition to the CSC. Only the CSC had a dedicated 24/7 stroke call team, and all EVT referrals were centralised to this centre.

Western Norway (10 hospitals), comprising Rogaland, Vestland and parts of Møre and Romsdal, served approximately 1.42 million inhabitants in 2024 and accounted for 1443 registered acute stroke cases in the Norwegian Stroke Registry. The region had 2 EVT centres with full 24/7 imaging and advanced protocols. Among 8 other stroke units, 3 had technical capacity for IVT in wake-up stroke and IVT in the extended-window, but only 2 applied these routinely. Five units lacked CTP, and none but the EVT centres had acute MRI (Figure 3). One CSC had a dedicated 24/7 stroke call team, while no stroke unit had such coverage; off-hours decisions were made by general on-call consultants.

South-Eastern Norway (22 hospitals), serving approximately 3.1 million inhabitants, represented the largest catchment area and recorded 5022 acute stroke admissions in 2024. The region is served by 4 EVT centres (2 co-located, 1 nearby and one ~ 300 km away with weekday-only EVT) and 18 stroke units, 3 of which transfer all acute patients to a CSC. Two of the CSCs have a dedicated 24/7 stroke call team. In South-Eastern Norway, 19 hospitals provided local acute stroke imaging, while 3 hospitals transferred all acute patients directly to a CSC. Among the 19 hospitals, 13 had CTP and 6 lacked it (Figure 3). Acute MRI was available in 12 hospitals, including 2 with weekday-only access, while the remaining 7 had no acute MRI availability. Advanced protocol use was inconsistent, often limited by radiologist availability, and several units operated only during standard hours, requiring off-hours transfers.

### Imaging capabilities

CTA was nearly universal nationwide. CTP was available in 28 hospitals (56%), with substantial gaps in Western and Northern Norway. Acute MRI was available in 17 hospitals (34%), and even where scanners were available, 24/7 access was rare, particularly outside CSCs.

### Regional stroke networks

Organisation varied considerably, referring to formal structures for coordination, protocol development and quality improvement rather than day-to-day clinical collaboration:

- Central Norway had the most developed network with regular meetings, protocol harmonisation, quality review and emergency medical services integration.
- Northern Norway had no active formal network; previous annual multidisciplinary meetings ceased in 2024 due to loss of funding.
- South-Eastern Norway maintained a formal regional advisory board since 2015, overarching all 22 hospitals; this body was instrumental in developing EVT services. Activity declined after 2019 but was reactivated in 2024. In parallel, several smaller collaborative groups of 2–3 hospitals function as sub-regional stroke networks.
  Western Norway had no formal network at the time of assessment, though planning was underway.

The structure, funding and activity level of regional stroke networks are summarised in Table 2.

**Table 2:** Organisation and maturity of regional stroke networks in Norway.

Region	Network status	Key activities	Frequency of meetings	Funding score	Notes
**Northern Norway**	Inactive since 2024	Annual multidisciplinary meetings (ceased)	—	Previously funded regionally	Loss of funding stopped meetings
**Central Norway**	Formal, mature	Protocol harmonisation, QI, EMS coordination	4/year	Regional authority	Only region with full participation
**Western Norway**	None	—	—	—	Discussions underway
**South-Eastern Norway**	Formal advisory board since 2015	EVT model development, network coordination	Variable	Regional authority	Reactivated 2024

Each health region is described according to the existence and status of a formal stroke network, core activities, meeting frequency and funding source. “Funding score” refers to the presence and stability of secure financial support from regional health authorities. Notes summarise relevant contextual information for network functioning and changes over time.

## Discussion

This national survey of all 50 Norwegian hospitals providing acute stroke care reveals substantial within-country regional variation in both the availability and implementation of advanced reperfusion treatments. By integrating hospital-level structural data with population-based data from the Norwegian Stroke Registry, our findings demonstrate how organisational capacity and geography jointly shape access to timely reperfusion treatment. While IVT was universally available within the 0–4.5 h time window, approximately half of the hospitals routinely delivered the full combination of IVT < 4.5 h, IVT in wake-up stroke and IVT in the extended-window. EVT was provided in 8 hospitals, with uneven geographic distribution, leaving large geographic areas without direct access, necessitating interhospital transfers over long distances. Crucially, we identified a consistent gap between technical capacity and routine clinical use—often linked to limited staffing, lack of 24/7 imaging access or absence of formalised protocols.

Advanced imaging capacity was uneven. CTA was almost universal, but CTP was unavailable in 44% of hospitals and acute MRI in 66%, with the lowest coverage in Western Norway and Northern Norway. Even where scanners were available, out-of-hours access was uncommon outside CSCs, and 2 hospitals offered acute MRI only during weekdays. These regional differences may also reflect longer prehospital transport distances and more dispersed populations, particularly in Northern Norway.

Over the last decade, the introduction of EVT, IVT in the extended time window and imaging-based patient selection has transformed acute stroke care, with demonstrable improvements in functional outcomes.^4–7^ A major challenge now is ensuring equitable access to these treatments. Our findings confirm that the presence of technical infrastructure does not guarantee its use—a phenomenon also reported in other European countries.^13^

In Norway, several hospitals possessed CTP or MRI capabilities, but did not use them for acute stroke decision-making outside daytime hours. This limits the application of advanced protocols, especially in rural areas where patient transfer to CSCs may be delayed. The result is avoidable missed opportunities for reperfusion therapy, which may contribute to regional disparities in patient outcomes.

Regional stroke networks varied markedly in organisational maturity, with clear consequences for service delivery. Central Norway’s well-funded, active network—with designated clinical leads, structured meetings and integration with the CSC and emergency medical services—facilitated uniform implementation of advanced reperfusion protocols across all stroke units. In contrast, Northern Norway’s suspension of network activities in 2024 following loss of dedicated funding showed how fragile such systems can be when dependent on discretionary budgets. South-Eastern Norway’s formal advisory board, highly influential in developing EVT pathways between 2015 and 2018, illustrates the potential for organisational structures to drive change; its decline with leadership turnover and subsequent reactivation in 2024 highlight both the vulnerability and renewability of regional coordination efforts. These examples underline how structural and organisational differences, despite national guidelines, contribute directly to unequal access to advanced stroke treatment.

These organisational gaps have both medical and economic consequences. Medically, delayed or absent reperfusion therapy is associated with worse functional outcomes, higher rates of institutionalisation and greater long-term disability.^14^^,^^15^ The total cost of stroke in Europe in 2017 is calculated to €60 billion/year.^16^ The economic burden is projected to rise to €86 billion by 2040 without urgent reform.^17^ Although per-patient treatment costs vary considerably between countries, the resources to sustain regional coordination—regular meetings, protocol harmonisation and continuous data review—are modest in comparison.

Our findings directly address 3 research priorities from the Stroke Action Plan for Europe (SAP-E)^12^:

1. Within-country and regional variation

This study systematically documents hospital-level variation in both technical capability and protocol implementation across health regions. Such granularity is rarely available in national reporting, yet it is essential to identify inequities that may be masked by aggregated national averages.

1. Strengthening regional stroke networks

The contrast between regions with and without formal networks supports the SAP-E emphasis on regional coordination as a driver of quality improvement. Our findings suggest that network maturity correlates with protocol implementation, providing empirical support for this policy direction.

1. Developing comparable data systems

The classification framework used here—combining treatment availability, imaging capacity and organisational maturity—could be adapted for use in other countries. Standardising such data collection would facilitate cross-country comparisons and benchmarking, a key SAP-E goal.

Our findings are consistent with European studies showing that even within high-income countries offering universal health care, inequities in access to reperfusion therapies and advanced imaging continue to persist.^13^^,^^18^^,^^19^ By linking these inequities to differences in regional network maturity, our work offers a new lens for understanding and addressing them.

### Call to action

To close the gap between technical capacity and routine delivery, we propose:

- Institutionalising regional stroke networks with secure funding, formal mandates and accountability to health authorities.
- Guaranteeing 24/7 access to key imaging modalities (CTA, CTP, MRI) and stroke specialist decision-making, especially in non-CSC hospitals.
- Integrating the hospital classification system from this study into national quality reporting, enabling continuous monitoring of regional disparities.
- Targeted quality improvement programmes in all regions, addressing the specific gaps between capacity and practice identified in each.

These measures are low-cost relative to the substantial health and economic burden of untreated stroke. Ensuring that all patients—regardless of geographic location—have access to the full range of evidence-based acute stroke treatments is both a medical imperative and a core goal of the SAP-E vision.

## Data Availability

The dataset generated from the national survey of hospitals is available from the corresponding author upon reasonable request.
